# Exploring young adults’ e-cigarette use behavior during COVID-19

**DOI:** 10.18332/tpc/155332

**Published:** 2022-12-15

**Authors:** Michelle Clausen, Katelyn F. Romm, Carla J. Berg, Annie C. Ciceron, Caroline Fuss, Breesa Bennett, Daisy Le

**Affiliations:** 1 Department of Policy, Populations, and Systems, School of Nursing, George Washington University, Washington, United States; 2 TSET Health Promotion Research Center, Stephenson Cancer Center, University of Oklahoma Health Sciences Center, Oklahoma City, United States; 3 Department of Pediatrics, College of Medicine, University of Oklahoma Health Sciences Center, Oklahoma City, United States; 4 Department of Prevention and Community Health, Milken Institute School of Public Health, George Washington University, Washington, United States; 5 George Washington Cancer Center, George Washington University, Washington, United States; 6 Department of Epidemiology, Milken Institute School of Public Health, George Washington University, Washington, United States

**Keywords:** cannabinoids, cannabidiol, prevention, risk perceptions, public health policy, young adults

## Abstract

**INTRODUCTION:**

Changes in daily life related to COVID-19 have impacted e-cigarette use, particularly in young adults. This cross-sectional mixed-methods study explored young adults’ perceptions regarding how COVID-19 influenced their e-cigarette use.

**METHODS:**

We analyzed Fall 2020 survey data from 726 past 6-month e-cigarette users (mean age=24.15 years, 51.1% female, 35.5% sexual minority, 4.4% Black, 10.2% Asian, 12.1% Hispanic) and Spring 2021 semi-structured interview data among a subset of 40 participants (mean age=26.30 years, 35.0% female, 45.0% sexual minority, 5.0% Black, 22.5% Asian, 12.5% Hispanic). Participants were drawn from 6 metropolitan statistical areas with varied tobacco and cannabis legislative contexts.

**RESULTS:**

Among survey participants, 44.4% also smoked cigarettes, 54.0% other tobacco products, and 60.1% used cannabis. They reported various changes in their daily lives, including changes in the nature and/or status of employment (e.g. 15.3% were laid off, 72.8% experienced household income loss). Regarding changes in e-cigarette use since COVID-19, 22.6% tried to cut down and 16.0% tried to quit. Interview participants commonly indicated that they increased their use due to stress, boredom, changes in accessibility, and/or changes to daily environment that made e-cigarette use more feasible.

**CONCLUSIONS:**

Results highlight the importance of promoting opportunities for young adults to build relationships to decrease stress, foster a sense of belonging, and increase quality of life (e.g. increasing the accessibility to mental health and social support services, intentionally engaging young adults in pandemic-appropriate community-building and extracurricular activities). This research may help to inform future e-cigarette cessation interventions that consider the unique challenges of societal stressors, such as pandemics.

## INTRODUCTION

Between 2017 and 2019, the use of e-cigarettes, which typically involves e-liquids containing nicotine, doubled among young adults in the US^1^. Despite the public health toll of e-cigarette use, use among young adults continues to increase^1,2^. This is a concern as young adults who use e-cigarettes are more likely to use other substances, including other tobacco and cannabis^1^. Research has highlighted the adverse effects of nicotine and other hazardous chemicals included in e-cigarettes on the developing brain and nervous system^2^. Due to the short- and long-term health effects associated with e-cigarette use, prevention efforts and cessation strategies that target young adults are critical^1^.

The novel coronavirus pandemic (COVID-19) has provided unique insights regarding e-cigarette and other substance use behavior among young adults^3^. Understanding how the pandemic has altered e-cigarette and other substance use in this population is important, as it has implications for addressing use post-pandemic and for confronting use during other societal stressors. For example, during the pandemic, various states issued non-essential business closures and put forth mandated stay-at-home orders which altered the daily life of many young adults^3^. Although the timing and duration of pandemic-related restrictions varied, these closures also limited access to e-cigarette products in some jurisdictions, thus influencing the use of e-cigarettes by young adults^4^.

In addition, current evidence suggests that both combustible and electronic tobacco products cause damage to the lungs^5-7^ and alter the immune system’s response, leading to increased susceptibility to most respiratory viruses including COVID-19^7^. Accordingly, during the pandemic, states issued advisories regarding the potential increased risk of spreading COVID-19 and exacerbation of COVID-19 symptoms among those who smoke tobacco and use e-cigarettes^8^, and many experts recommended tapering or eliminating the use of these products during the pandemic^3,8^. Further investigation of tobacco and e-cigarette use as a risk factor for severe disease from COVID-19 is important for the development of preventative strategies^9^.

Research regarding e-cigarette use during the pandemic has been mixed^10,11^. Early studies initially reported decreased e-cigarette use among young adults but cited increases in tobacco and other substance use^7,12^. As the pandemic continues, contradictory evidence has emerged, challenging these initial findings, and suggesting an increase in e-cigarette use during the pandemic^9,13^.

Given the changes in daily life for young adults introduced by the pandemic, more in-depth knowledge is necessary to understand how COVID-19 has changed e-cigarette use and how the pandemic provided opportunities for changes in e-cigarette use behaviors. This information is critical to inform the development of affordable, accessible, effective, and culturally congruent solutions aimed at reducing e-cigarette and other substance use during a future pandemic or societal stressor that might entail long-term disruptions to daily life. Thus, this cross-sectional mixed-methods study: 1) examined COVID-19 related factors associated with young adult e-cigarette use; and 2) explored young adults’ perceptions regarding how COVID-19 influenced their e-cigarette use.

## METHODS

### Study overview

The current study analyzed cross-sectional quantitative and qualitative data from young adults (aged 18–34 years) participating in the ‘Vape shop Advertising, Place characteristics and Effects Surveillance (VAPES)’ study, which examines the US vape retail environment and its impact on e-cigarette use. This 2-year, 5-wave longitudinal cohort study was launched in Fall 2018 and entailed biannual assessments until Fall 2020. Participants were drawn from 6 metropolitan statistical areas (MSAs: Atlanta, Boston, Minneapolis, Oklahoma City, San Diego, and Seattle) with varied tobacco and cannabis legislative contexts^14^. This study is described in detail elsewhere^15^.

Ads posted on social media (i.e. Facebook, Reddit) recruited eligible individuals (i.e. aged 18–34 years, residing in one of the 6 aforementioned MSAs as indicated by residential zip code, English speaking). Individuals who clicked on ads were directed to a webpage with a study description and consent form and screened for eligibility. Purposive, quota-based sampling ensured sufficient representation of approximately one-third of e-cigarette and cigarette users, respectively, roughly equal numbers of men and women, and 40% of racial/ethnic minority. Recruitment was capped by MSA.

Individuals allowed to advance to the study (i.e. eligible and still being recruited in the respective MSA) were routed to complete the online Wave 1 (W1) survey (administered via Alchemer). Upon completion of the survey, participants were notified that they would be asked to confirm their participation by clicking a ‘confirm’ button included in an email 7 days later. Once participants clicked ‘confirm’, they were officially enrolled in the study and emailed their first incentive in the form of a $10 Amazon electronic gift card.

Of the 10433 Facebook and Reddit users who clicked on ads, 9847 consented, of which 2751 (27.9%) were not allowed to advance because they were either ineligible (n=1472) and/or excluded to reach subgroup target enrollment (n=1279). Of those allowed to advance to the survey, the proportion of completers versus partial completers was 48.8% (3460/7096) versus 51.2% (3636/7096). Partial completers were deemed ineligible for the remainder of the study; the majority of partial completers (n=2469; 67.9%) completed only the initial sociodemographic section of the survey. Of the 3460 who completed the W1 survey, 3006 (86.9%) confirmed participation^15^.

### Quantitative data collection

Current quantitative analyses used sociodemographic data from W1 (Fall 2018, n=3006) and assessments of e-cigarette use characteristics and COVID-19 related factors among young adult e-cigarette users from W5 (Fall 2020, n=2476; 726 of whom were past 6-month e-cigarette users). W5 MSA data were reported to account for movement.

### Measures

*Sociodemographic and related factors during COVID-19*

At W1, participants reported their age, gender, race, ethnicity, and sexual orientation. At W5, we also assessed their college enrollment, household composition, employment status, and nature of employment during COVID-19 (e.g. working outside of home, working from home, laid off, not working), loss of income or employment among participants and household members, and whether they were under state orders restricting activity, e.g. stay-at-home orders (Table 1).

**Table 1:** Characteristics and COVID-19 related factors among survey participants who reported past 6-month e-cigarette use and a subset of past 30-day survey participants who participated in interviews across 6 metropolitan statistical areas in the US

*Characteristics*	*Survey participants reporting past 6-month e-cigarette use (2020) (n=726) n (%)*	*Past 30-day e-cigarette users participating in interview (2021) (n=40) n (%)*
**MSA**		
Atlanta	102 (14.0)	8 (20.0)
Boston	110 (15.2)	5 (12.5)
Minneapolis	134 (18.5)	10 (25.0)
Oklahoma City	77 (10.6)	3 (7.5)
San Diego	106 (14.6)	5 (12.5)
Seattle	131 (18.0)	6 (15.0)
Other	58 (8.0)	2 (5.0)
**Age** (years), mean ± SD	24.15 ± 4.84	26.30 ± 4.39
Female	371 (51.1)	14 (35.0)
Sexual minority	258 (35.5)	18 (45.0)
**Race**		
White	534 (73.6)	23 (57.5)
Black	32 (4.4)	2 (5.0)
Asian	74 (10.2)	9 (22.5)
Other	86 (11.8)	6 (15.0)
Hispanic	88 (12.1)	5 (12.5)
**Education level**		
Bachelor's or higher	453 (62.4)	30 (75.0)
Enrolled in college during 2020–2021 academic year	250 (34.4)	10 (25.0)
**Household composition**		
No one else	77 (10.6)	6 (15.0)
Roommates/friends	217 (29.9)	11 (27.5)
Parents/guardians	173 (23.8)	10 (25.0)
Siblings	105 (14.5)	5 (12.5)
Spouse/romantic partner	319 (43.9)	18 (45.0)
Children	108 (14.9)	4 (10.0)
Extended family	24 (3.3)	1 (2.5)
Other	9 (1.2)	0 (0)
**Impact of COVID-19 on employment** (part-time or full-time)		
Not working before COVID-19	109 (15.0)	6 (15.0)
Working outside the home and still going to work	198 (27.3)	13 (32.5)
Working outside the home but now working from home	135 (18.6)	9 (22.5)
Working from home and still working from home	34 (4.7)	1 (2.5)
Laid off from a job	111 (15.3)	8 (20.0)
New employment as a result of COVID-19 associated changes	95 (13.1)	3 (7.5)
**Change in employment/income since news of COVID-19**		
Personal loss of employment/main income source	197 (27.1)	10 (25.0)
Personal loss of some income but not major source	147 (20.2)	8 (20.0)
Loss of household member’s employment/main income source	116 (16.0)	8 (20.0)
Loss of some household member’s income but not major source	69 (9.5)	4 (10.0)
Took on more responsibility for caring of children	79 (10.9)	2 (5.0)
Household started to have more financial problems	219 (30.2)	13 (32.5)
**Other experiences due to COVID-19**		
No restrictions (normal daily life)	281 (38.7)	23 (57.5)
Sheltering in place	235 (32.4)	14 (35.0)
Lockdown (people living in my area cannot leave their homes)	52 (7.2)	0 (0)
Self-quarantine (voluntarily due to risk of getting COVID-19)	208 (28.7)	11 (27.5)
Quarantine (required due to risk of getting COVID-19)	38 (5.2)	1 (2.5)
Living with someone who tested positive for COVID-19	15 (2.1)	1 (2.5)
Diagnosed as positive with COVID-19	7 (1.0)	1 (2.5)
Know someone who died because of COVID-19	110 (15.2)	2 (5.0)

MSA: metropolitan statistical area. Sociodemographic data from W1 (Fall 2018; n=3006) and assessments of COVID-19 related factors among young adult e-cigarette users from W5 (Fall 2020; n=2476; 726 of whom were past 6-month e-cigarette users). W5 MSA data were reported to account for movement.

*E-cigarette use and related factors during COVID-19*

At W5, participants were asked, ‘In the past 6 months, on how many days have you used e-cigarettes?’ (0=not at all; 5=almost daily, or daily). Past 6-month users were also asked to report the nature of their e-cigarette use (i.e. most common e-liquid flavors used, use of nicotine salt, device type). We also asked, ‘During COVID-19, did you experience any of the following: you tried to cut down on vaping; you tried to quit vaping; you stocked up on vaping products, such as e-liquids; you worried your vape shop would close or go out of business; you worried your source of vaping products (not from a vape shop) would be difficult to access; your vape shop went out of business; your source of vaping products (not from a vape shop) was difficult to access; you noticed more options for home delivery of vaping products.’

### Qualitative data collection

Qualitative data collection and analyses were guided by the COREQ (Consolidated Criteria for Reporting Qualitative Research) guidelines^16^. In February–April 2021, participants who reported past 30-day e-cigarette use at W5 were recruited via email to participate in semi-structured interviews. To participate in interviews, the only eligibility criterion was reporting past 30-day use on the eligibility screener during the recruitment period; however, we purposively sampled e-cigarette users with representation across the sexes, sexual orientation, and racial/ethnic backgrounds.

Of the 139 participants recruited via email, 105 (75.5%) began the eligibility screener, of whom 11 (10.5%) partially and 94 (89.5%) fully completed the screener. Of the 94, 34 (36.2%) were not eligible, and 60 (63.8%) were eligible and consented. Of the 60, 40 (66.7%) were successfully scheduled for and participated in an interview, at which point, saturation had been reached.

The semi-structured interview guide was developed by the study team to explore experiences with tobacco product use. The initial interview guide was piloted through mock interviews among a sample of 4 graduate research assistants, wherein we tested question phrasing, clarity, and necessary probes. The interview guide was revised after the first 3 interviews to ensure clarity and comprehensiveness. Interview guide topics explored in the current analyses focused on how participants’ e-cigarette use changed during COVID-19 and if they made any attempt to quit or cut back on vaping during this time.

We conducted interviews via WebEx after obtaining online consent via email. The interviews, which lasted about 45 minutes, were facilitated by 4 female graduate research assistants trained in qualitative data collection. The interviews were audio-recorded for subsequent coding. Participants were debriefed and compensated with a $35 Amazon e-gift card upon interview completion. All audio-recorded interviews were uploaded to a secure, password-protected computer, and the recordings were transcribed verbatim by a contracted professional transcription service.

### Statistical analysis

Survey data were analyzed descriptively to characterize participants, using SPSS 24.0. Qualitative data were analyzed using thematic analyses. Transcripts were systematically coded using QRS Software NVivo v12 and cross-checked for agreement regarding the application of the codes. Team discussions occurred regularly in which codebook themes were redefined, inclusion and exclusion criteria were set, and representative passages were identified. Discrepancies regarding code choices were resolved through discussion in a process of constant comparison and until consensus was reached (kappa=93.3%). Inter-rater reliability was calculated for each code through use of an intra-class correlation coefficient and was deemed acceptable if the coefficient was ≥0.80. Content codes were used to thematically group similar interview text; themes were organized into overarching domains compiled with representative quotations, which were edited for readability. Balancing the controversy in qualitative research regarding whether to quantify qualitative results, we indicated the frequency with which themes were provided by participants by quantizing them as ‘most’, ‘many’, ‘almost half’, ‘some’, and ‘a few’^16^.

### Ethical approval

All human participants were treated in accord with the Principles of the Ethical Practice of Public Health of APHA, gave their informed consent to participate, and the study was reviewed and approved by the Institutional Review Boards of Emory University and George Washington University [interviews; NCR203050].

## RESULTS

### Quantitative results

This sample of past 6-month e-cigarette users at W5 (n=726) had an average age of 24.15 (SD=4.84) years, 51.1% were female, 35.5% sexual minority, 73.6% White, 4.4% Black, 10.2% Asian, and 12.1% Hispanic (Table 1). Among past 6-month e-cigarette users at W5, 34.4% were enrolled in college. Most lived with a spouse/romantic partner (43.9%), roommates/friends (29.9%), or with parents (23.8%). Regarding employment, 15.0% reported not working before the pandemic. When asked about the impact of COVID-19 on employment, 27.3% continued working outside the home, 18.6% changed to working from home, and 15.3% were laid off. Relatedly, 72.8% indicated a personal loss or loss of a household member’s income and 30.2% indicated more household financial problems. At W5, 38.7% indicated no COVID-related restrictions, 32.4% reported sheltering in place, 7.2% were experiencing a lockdown, and 28.7% were in self-quarantine.

As shown in Table 2, 616 (84.8%) of W5 past 6-month e-cigarette users reported past 30-day e-cigarette use and an average of 14.97 (SD=12.84) days of use in the past 30 days; past 30-day users used e-cigarettes 17.51 days (SD=12.17). Roughly half reported past 30-day use of cigarettes (44.4%), other tobacco (54.0%), and cannabis (60.1%). Notably, 22.6% reported attempts to cut down on vaping, 16% tried to quit vaping, 23.7% stocked up on vaping products during COVID-19, several worried their product source would close (19.6% regarding vape shops, 15.3% other sources), some reported their source closed (5.5% regarding vape shops, 7.6% other sources), and 8.8% noticed more home delivery options.

**Table 2:** E-cigarette use and related experiences during COVID-19 among survey participants who reported past 6-month e-cigarette use and a subset of past 30-day survey participants who participated in interviews across 6 metropolitan statistical areas in the US

*Variables*	*Survey participants reporting past 6-month e-cigarette use (2020) (n=726) n (%)*	*Past 30-day e-cigarette users participating in interview (2021) (n=40) n (%)*
**E-cigarette use**		
Any use in past 30 days	616 (84.8)	40 (100.0)
Days of use in past 30 days among past 6-month users, mean ± SD	14.97 ± 12.84	27.63 ± 4.97
**Flavors most commonly used** (list up to 3)*		
Tobacco	98 (13.5)	8 (20.0)
Menthol or mint	356 (49.0)	23 (57.5)
Fruit flavors	502 (69.1)	33 (82.5)
Caramel, vanilla, chocolate, cream	85 (11.7)	5 (12.5)
Candy flavors	196 (27.0)	14 (35.0)
**Typically use nicotine salt**		
Never	290 (39.9)	14 (35.0)
Rarely	118 (16.3)	3 (7.5)
Some of the time	102 (14.0)	5 (12.5)
Most of the time	77 (10.6)	5 (12.5)
All of the time	139 (19.1)	13 (32.5)
Rechargeable (vs disposable) device use	545 (75.1)	35 (87.5)
Open (vs closed) system/tank use	365 (50.3)	24 (60.0)
**Other tobacco/substance use, past 30-days**		
Cigarettes	322 (44.4)	22 (55.0)
Other tobacco products	392 (54.0)	12 (30.0)
Cannabis	436 (60.1)	18 (45.0)
**Changes in e-cigarette use/access since COVID-19**		
Tried to cut down on vaping	164 (22.6)	8 (20.0)
Tried to quit vaping	116 (16.0)	3 (7.5)
Stocked up on vaping products, such as e-liquids	172 (23.7)	13 (32.5)
Worried vape shop would close or go out of business	142 (19.6)	13 (32.5)
Worried vaping product source (not vape shop) difficult to access	111 (15.3)	10 (25.0)
Vape shop went out of business	40 (5.5)	4 (10.0)
Vaping product source (not a vape shop) difficult to access	55 (7.6)	4 (10.0)
Tried to cut down on vaping	164 (22.6)	8 (20.0)
Tried to quit vaping	116 (16.0)	3 (7.5)
Noticed more options for home delivery of vaping products	64 (8.8)	3 (7.5)

* Survey sample also reported: coffee or tea 35 (4.8%), alcoholic drink flavors 26 (3.6%), other food flavors 84 (11.6%), other 17 (2.3%), none of these 37 (5.1%).

Assessments of e-cigarette use characteristics among young adult e-cigarette users from W5 (Fall 2020, n=2476; 726 of whom were past 6-month e-cigarette users). W5 MSA data were reported to account for movement.

### Qualitative results

Semi-structured interview participants (n=40) had an average age of 26.30 (SD=4.39) years, 35.0% were female, 45.0% sexual minority, 57.5% White, 5.0% Black, 22.5% Asian, and 12.5% Hispanic (Table 1). The proportions residing across the MSAs ranged from 7.5% in Oklahoma City to 25.0% in Minneapolis, with 5.0% residing outside the 6 MSAs (due to moving since W1). Table 3 presents themes, and selected quotes regarding: 1) the impact of COVID-19 on the accessibility of product; 2) stress; 3) boredom; and 4) changes in daily environment.

**Table 3:** A subset of past 30-day survey participants who participated in interviews describe experiences with and attitudes toward e-cigarette, tobacco, and other substance use during the COVID-19 pandemic, 2021 (N=40)

*Theme*	*Representative quote*
**The impact of COVID-19 on accessibility**	‘All those same places that I would buy e-cigarettes were closing, and I was temporarily laid off. So, there was no way that if I got 10 bucks, that I was going to go waste it on vaping juice.’ (e-cigarette and cannabis user, Seattle) ‘I had less access to it, and I just wasn't in the right mental state to use it.’ (e-cigarette user, Boston)
**Stress**	‘It increased a lot. I lost my first job during this pandemic. Then I had to go hunting for a new job, and a lot of them just didn't work out. So it's been a lot of job hopping, and that's very, very stressful.’ (e-cigarette user, Oklahoma City) ‘I definitely increased vaping. I smoke to relieve stress because this has been a very stressful year.’ (e-cigarette, cigarette, cannabis, and CBD user, Minneapolis)
**Boredom**	‘I am bored more often during COVID, so I have more opportunities to vape.’ (e-cigarette user, San Diego) ‘You're just stuck at home, nothing to do, so I started vaping more.’ (e-cigarette cigarette user, Atlanta)
**Changes in daily environment**	‘It's probably increased during the pandemic, partially because I am at home all the time now and I vape freely in my home even when my boyfriend is trying to quit.’ (e-cigarette, cigarette, cannabis, and CBD user, Minneapolis) ‘I would say slight increase in vaping, mostly because I want to do something. For now, it's sitting at home and spending a lot more time at home, supplanted some of the other social things I would have done out of the house with other people.’ (e-cigarette and cannabis user, Minneapolis) ‘So I was working from home, and could vape literally all day long at my desk, instead of being somewhere where in my office prior to having to work from home.’ (e-cigarette, cigarette, and cannabis user, Atlanta)

*The impact of COVID-19 on accessibility of product*

Many participants reported accessibility issues related to e-cigarette products and other substances during the pandemic, and many reported a decrease in e-cigarette use due to limited access to products. Many participants indicated stocking up on flavored e-liquids or obtaining flavors from alternative sources due to business closures and limitations in supply brought about by COVID-19. One participant described the impact of vape stores closing on access to e-cigarette products during the pandemic:

‘*When COVID-19 first started, everything was starting to shut down and so I stocked up.*’ (e-cigarette user, Seattle)

Store closures encouraged some young adult e-cigarette users to change their purchasing habits which included purchasing products through new avenues such as the Internet.

*Stress*

Some participants reported increased stress during the pandemic, which influenced their e-cigarette and other substance use. One participant reported that stress due to work schedule changes:

*‘… doubled during the pandemic’* (e-cigarette, cigarette, and cannabis user, Minneapolis)

One participant described the stress related to job loss at the beginning of the pandemic, which prompted an increase in e-cigarette use. In addition, stress due to major life changes such as job loss prompted increases in vaping during the pandemic:

*‘It increased a lot. I lost my first job during this pandemic. Then I had to go hunting for a new job, and a lot of them just didn’t work out. So it’s been a lot of job-hopping, and that’s very stressful.’* (e-cigarette user, Oklahoma City)

*Boredom*

As a result of daily life changes brought about by the pandemic, many participants found more opportunities to vape:

*‘A good part of me wanted to vape all the time, so if I went into a different room, or if I got bored, I hit my vape.’* (e-cigarette and cannabis user, Boston)

*Changes in daily environment*

Most participants reported an increase in e-cigarette use during the pandemic due to significant changes in daily social interactions. Social distancing restrictions and stay-at-home orders imposed by COVID-19 kept many young adults at home, and most participants who increased their e-cigarette use during the pandemic noted that they had more occasions to use e-cigarettes. In addition, school closures and teleworking schedules provided a unique opportunity to use e-cigarettes freely without constraints at home:

*‘It’s probably increased during the pandemic, partially because I am at home all the time now and I vape freely in my home even when my boyfriend is trying to quit.’* (e-cigarette, cigarette, cannabis, and CBD user, Minneapolis)

Some participants noted that changes in daily work environments, such as a transition to telework, increased freedom to engage in vaping:

*‘So I was working from home, and could vape all day long at my desk, instead of being somewhere in my office before having to work from home.’* (e-cigarette, cigarette, and cannabis user, Atlanta)

Pre-pandemic e-cigarette use was limited by restrictions in work or school settings; however, the pandemic left many participants homebound. This dramatic change in life provided ample time and setting to allow for unrestricted e-cigarette use:

*‘So when I would go into school, we’re not supposed to vape, so definitely being able to do it freely all day long has increased my usage.’* (e-cigarette, cigarette, cannabis, and CBD user, Minneapolis)

Changes in social interactions brought upon by COVID-19 provided an opportunity for young adults to initiate e-cigarette use. One participant noted that the lack of socialization aided in reduced e-cigarette use:

*‘It’s starting to reduce just because I have a little one and I’m not going out as much as I used to. So, it’s starting to decrease.’* (e-cigarette and cannabis user, Minneapolis)

Another young adult e-cigarette user described how being homebound forced reflection on use habits, which led to an overall reduction in use:

*‘Honestly, I’ve become more aware of it just because I’m in the same room all day. If I get bored and hit my vape but I am in my room all day… [I’m] forced to reflect on how much I vape.’* (e-cigarette, cigarette, cannabis, and CBD user, Boston)

## DISCUSSION

This study underscores the impact of COVID-19 on young adults’ e-cigarette use, particularly via changes in accessibility to vaping products due to retail disruptions and state-order business closures, stress, boredom, and changes in the daily environment. Quantitative findings suggested that roughly one-fifth of participants made attempts to cut down or quit vaping (respectively), and some interview participants reported decreasing their use, reflecting literature indicating a decrease in the use of current e-cigarette use among young adults^5,7,8,12^.

Most young adults in the qualitative interviews indicated increases in e-cigarette use during the pandemic, which may be likely partially attributable to the fact that interview participants were those who continued to use e-cigarettes during the pandemic (i.e. as part of the eligibility criteria). Several factors may have driven decreases in e-cigarette use, or increased attempts to cut down or quit. For example, about a quarter of participants reported living at home with parents or guardians, which is associated with less e-cigarette use^5,7,8,12^. Additionally, others worried about the impact of vaping on their lung function, particularly during COVID-19, and some experienced disruptions in e-cigarette supply and demand^5,7,8,12,17^. Specifically, a proportion of e-cigarette users were worried that their source of vaping products would close, and some had their source go out of business. However, others reported stocking up on vaping products since COVID-19, which is consistent with the varied conclusions in the literature regarding e-cigarette use during the pandemic^7,9,12,13^. However, most participants in this study were in their mid twenties and may have experienced different pandemic-related challenges than a younger population.

Increases in e-cigarette use were often attributed to COVID-19’s impact on daily life, consistent with recent research^9,13^. For example, some participants indicated COVID-19 related changes in employment – from originally working outside the home to now working from home – or being laid off^18^. Additionally, shelter-in-place orders, mandated lockdowns, and quarantining (either voluntarily or required) forced many young adults to be homebound, free from vaping and smoking limitations^18,19^. These experiences may be a result of increased boredom and stress, including stress triggered by the unknown trajectory of the virus.

### Limitations

Limitations include limited generalizability to young adults across the US due to the sampling strategy (e.g. recruiting a sample consisting of about one-third cigarette and e-cigarette users, via social media). Additionally, participants are likely to differ from those who chose not to participate regarding some important characteristics (e.g. preconceived positive attitudes towards screening and health-conscious behaviors). However, both for the parent study and the interview component of the study, we purposively sampled to obtain the participation of individuals of varying age, sex, and race/ethnicity, and these 6 MSAs represented a broad range of tobacco and e-cigarette policies as well as COVID-related state orders. Particularly noteworthy, the interviews were conducted among past 30-day e-cigarette users, and thus do not represent the perspectives of those who quit using e-cigarettes during the pandemic.

### Implications for policy and/or practice

Understanding the effects of the ongoing and evolving COVID-19 pandemic on young adult e-cigarette use is imperative^20,21^. Undoubtedly, the COVID-19 pandemic has presented young adults with unprecedented challenges pertaining to mental health and overall wellness. Results from this study and others have highlighted the importance of promoting opportunities for young adults to build relationships, particularly during a time in history when experiences of loneliness and isolation have escalated. Increasing the accessibility to mental health and social support services for young adults, and intentionally engaging them in pandemic-appropriate community-building and extracurricular activities, may decrease stress, foster a sense of belonging, and increase quality of life. Future prevention research efforts should continue to: 1) monitor young adults’ vaping behavior over the course of the pandemic; and 2) examine the relationship between the initiation of vaping, or increased vaping, during different stages of the pandemic on future substance use.

## CONCLUSIONS

Current findings highlight important COVID-19 related factors associated with e-cigarette use among young adults. Many individuals reported increased e-cigarette use during the pandemic as a result of COVID-19 related disruptions in product availability, stress, boredom, and changes in the daily environment. This research may help to inform future e-cigarette cessation interventions that consider the unique challenges of societal stressors, such as pandemics.

## Data Availability

The data supporting this research are available from the authors on reasonable request.
